# Vitamin D as an Immune Modulator in Systemic Lupus Erythematosus: A Narrative Review

**DOI:** 10.3390/life15101580

**Published:** 2025-10-10

**Authors:** Oana Raluca Predescu, Florentin Ananu Vreju, Stefan Cristian Dinescu, Cristina Elena Bita, Anca Emanuela Musetescu, Alesandra Florescu, Paulina Lucia Ciurea

**Affiliations:** 1 Doctoral School, University of Medicine and Pharmacy of Craiova, 200349 Craiova, Romania; raluca.predescu@gmail.com; 2 Department of Rheumatology, University of Medicine and Pharmacy of Craiova, 200349 Craiova, Romania; florentin.vreju@umfcv.ro (F.A.V.); stefan.dinescu@umfcv.ro (S.C.D.); cristina.gofita@umfcv.ro (C.E.B.); anca.musetescu@umfcv.ro (A.E.M.); paulina.ciurea@umfcv.ro (P.L.C.)

**Keywords:** systemic lupus erythematosus, vitamin D, disease activity, lupus nephritis, neurolupus, cardiovascular risk

## Abstract

Systemic lupus erythematosus (SLE) is a multisystem autoimmune disease in which environmental factors modulate genetically determined immune dysregulation. Vitamin D has emerged as a plausible modifier of disease expression because its active metabolite signals through the vitamin D receptor on innate and adaptive immune cells and influences antigen presentation, cytokine balance, and lymphocyte differentiation. This narrative review synthesizes current evidence on vitamin D status and supplementation in SLE with attention to organ-specific domains. Observational studies consistently report high rates of hypovitaminosis D in SLE and associations with less favorable clinical profiles, including higher global and renal disease activity, adverse cardiometabolic features, greater infection vulnerability, and neuropsychiatric manifestations. Preclinical models demonstrate neuroprotective and barrier-stabilizing actions of vitamin D analogs, supporting biological plausibility. Interventional trials indicate that supplementation safely corrects deficiency and shows signals of benefit for selected outcomes (e.g., modest activity reductions or fatigue in specific contexts), although effects on interferon signatures, complement, and autoantibodies are heterogeneous and often limited. Overall, current evidence supports optimization of vitamin D status as a low-risk adjunct in comprehensive SLE care while highlighting the need for adequately powered, organ-focused randomized trials using standardized measurements and prespecified endpoints to define causality, therapeutic targets, and long-term safety.

## 1. Introduction

Systemic lupus erythematosus (SLE) is a chronic, immune-mediated disorder characterized by marked clinical heterogeneity and the potential involvement of multiple organ systems. The classic systemic autoimmune illness, SLE, is distinguished by its remarkable clinical heterogeneity and persistent relapsing–remitting activity. The condition can impact almost every organ system, and its severity can range from minor mucocutaneous involvement to potentially fatal multiorgan damage. Common clinical manifestations include musculoskeletal involvement with non-erosive arthritis or arthralgia, which affects up to 90% of patients; mucocutaneous features such as malar rash, discoid lesions, photosensitivity, oral or nasal ulcers, and alopecia; and constitutional symptoms like fatigue, fever, and weight loss. More severe presentations include neuropsychiatric lupus with seizures, psychosis, cerebrovascular events, or cognitive impairment; lupus nephritis, which affects almost half of patients during the course of the disease and manifests as proteinuria, hematuria, or progressive renal failure; and cardiopulmonary complications like pericarditis, myocarditis, pulmonary hypertension, or serositis. Autoimmune hemolytic anemia, leukopenia, lymphopenia, and thrombocytopenia are among the common hematologic abnormalities. When seen as a whole, this clinical variation highlights the intricacy of SLE etiology and the challenge of customizing treatment for various organ systems [1,2,3,4,5].

The presence of a broad range of autoantibodies, many of which are included in classification criteria and correlate with disease phenotype, is a key immunopathological characteristic of SLE. More than 95% of patients have antinuclear antibodies (ANAs), which have a high sensitivity but little specificity [6,7]. Anti-double-stranded DNA (anti-dsDNA) antibodies are relatively specific for SLE, and titers often parallel disease activity, especially in lupus nephritis. Despite only being found in 20–30% of patients, anti-Smith (anti-Sm) antibodies are quite specific. Anti-U1 ribonucleoprotein (anti-U1-RNP), anti-Ro/SSA, and anti-La/SSB are other autoantibodies linked to overlap syndromes, cutaneous lupus, and congenital heart block. Antiphospholipid antibodies, such as anticardiolipin, anti-β2 glycoprotein I, and lupus anticoagulant, increase the risk of thrombosis and obstetric morbidity. Anti-ribosomal P (related to neuropsychiatric lupus), anti-C1q (associated with the severity of lupus nephritis), and anti-histone antibodies (especially in drug-induced lupus) are other antibodies that have therapeutic significance. Both the diagnostic specificity and the variety of immunopathological symptoms seen in SLE are influenced by this large autoantibody repertoire [8,9,10,11,12,13].

In healthy individuals, immune responses are tightly regulated to recognize and eliminate harmful antigens while preserving self-tolerance. In contrast, the breakdown of these regulatory mechanisms in SLE leads to loss of tolerance and the development of aberrant autoimmune reactions, in which self-antigens become unintended targets of immune attack. A central feature of SLE is the emergence of T-cell and B-cell dysregulation, resulting in the production of a broad spectrum of autoantibodies and sustained systemic inflammation. Although extensive research has focused on uncovering the mechanisms underlying this process, the precise pathways leading from immune dysfunction to overt clinical disease remain incompletely understood. Among the environmental factors implicated, vitamin D deficiency has attracted particular attention due to its potential contribution to the onset and progression of several autoimmune diseases, including SLE [14].

Vitamin D is a secosteroid hormone traditionally recognized for its pivotal role in mineral homeostasis and the maintenance of skeletal integrity. More recent findings, however, have expanded its physiological significance, linking vitamin D to immune regulation, cardiovascular health, and the pathophysiology of chronic inflammation. Increasing evidence now suggests that insufficient vitamin D status may contribute not only to the development but also to the morbidity and mortality associated with a range of chronic conditions, including autoimmune diseases [15]. In modern societies, lifestyle-related factors—such as reduced sun exposure, dietary insufficiency, and altered environmental habits—have led to a notable rise in vitamin D deficiency. In parallel, the growing use of reliable laboratory assays for measuring serum 25-hydroxyvitamin D [25(OH)D] has improved recognition of the widespread prevalence of hypovitaminosis D, further emphasizing its clinical and public health relevance [16].

Historically, 1,25-dihydroxyvitamin D has been primarily recognized for its fundamental role in regulating bone and mineral metabolism. In recent years, however, increasing attention has shifted toward its “non-classical” actions, with accumulating evidence linking vitamin D to a broad spectrum of physiological processes beyond skeletal health. Vitamin D has an array of “non-classical” effects on several physiological systems in addition to its traditional function in calcium-phosphate balance and bone metabolism. By altering both innate and adaptive responses, it controls immune homeostasis; it affects cardiovascular function by affecting endothelial integrity, vascular stiffness, and renin-angiotensin system activity; it influences glucose and lipid metabolism by altering insulin sensitivity and pancreatic β-cell function; and it affects neurocognitive processes by having neuroprotective, anti-inflammatory, and neurotransmission-modulating effects. Moreover, vitamin D has been linked to general cardiometabolic risk profiles, cancer biology, muscular function, and reproductive health [14,17,18,19,20,21].

The observation that immune cells express the vitamin D receptor (VDR) has profoundly expanded our understanding of vitamin D biology. This finding suggests that vitamin D plays an active role in coordinating interactions between innate and adaptive immunity, positioning it as a key mediator within the immune system [22].

Deficiency of vitamin D, or hypovitaminosis D, has been increasingly documented across several autoimmune disorders, including type 1 diabetes mellitus, multiple sclerosis, rheumatoid arthritis, and systemic lupus erythematosus [17,23,24,25,26]. With respect to SLE specifically, multiple lines of evidence implicate vitamin D as a potential contributor to its pathogenesis. An expanding body of research has investigated the association between vitamin D status and SLE onset, its role in modulating disease activity and clinical course, and the possible therapeutic impact of supplementation [27].

Despite these advances, recent systematic reviews and meta-analyses examining vitamin D insufficiency in SLE and the efficacy of supplementation strategies have highlighted significant knowledge gaps. Current data remain inconclusive regarding the relationship between serum vitamin D levels and SLE disease activity, as well as the extent to which supplementation achieves immunomodulatory benefits. This inconsistency likely reflects the complexity of vitamin D metabolism, methodological variability across studies, and the limited number of large-scale, high-quality clinical trials conducted to date.

## 2. Vitamin D and the Immune System

### 2.1. Vitamin D Physiology

Vitamin D is a steroid-based vitamin that mainly regulates intestinal calcium absorption, promotes renal calcium reabsorption, and triggers the release of calcium and phosphate from bones by osteoclasts [28]. The bulk of vitamin D is created in the skin’s epidermal layer by ultraviolet B rays between 280 and 315 nm, which convert 7-dehydrocholesterol in the epidermis into cholecalciferol (vitamin D_3_) [29]. Humans only acquire a small amount of vitamin D from food. Cholecalciferol binds to the vitamin D-binding protein in the circulation and travels to the liver. It is hydroxylated there to 25-hydroxycholecalciferol [25-(OH)D_3_] by hepatic 25-hydroxylase. In the last processing stage, 25-(OH)D_3_ is converted to 1–25 dihydroxycholecalciferol (1,25-[OH]_2_D_3_) or calcitriol by the action of 1-hydroxylase in the renal cortices [30]. Blood calcium and phosphate levels, as well as parathyroid hormone (PTH), are key indicators of the tightly regulated hydroxylation process in the kidneys [31].

The physiologically inactive 24,25-(OH)_2_D_3_ is created when 1,25-(OH)_2_D_3_ stimulates the 24-hydroxylation of 25-(OH)D_3_ [32]. This serves as a check mechanism to prevent an overabundance of 1,25-(OH)_2_D_3_, which may otherwise result in high calcium and phosphate levels. By binding to the vitamin D receptors (VDRs), a class of nuclear receptors that bind to particular DNA sequences known as vitamin D-responsive elements (VDREs), and heterodimerizing with the retinoid X receptor (RXR), 1,25-(OH)_2_D_3_, an active form of vitamin D, initiates the downstream signaling mechanisms [33]. Following its binding to vitamin D-responsive elements (VDREs), the VDR–RXR heterodimer attracts a number of co-activator proteins, including mediator complexes, CBP/p300, and steroid receptor co-activator-1 (SRC-1), which modify chromatin by acetylating and methylating histones. More than 900 genes that control immunological responses, cell division, apoptosis, calcium-phosphate balance, and cell proliferation can have their transcription enabled or inhibited by these epigenetic changes [34,35].

Vitamin D can function through non-genomic routes in addition to these traditional genomic effects. Membrane-associated VDR and protein disulfide isomerase family A member 3 (Pdia3) are responsible for these quick reactions, which trigger second messenger cascades that include mitogen-activated protein kinases (MAPK), phospholipase C, and protein kinase C (PKC). Additionally, vitamin D signaling reduces NF-κB activation, which lowers the generation of pro-inflammatory cytokines, and interacts with the PI3K/AKT pathway, which supports cell survival and metabolism [35,36].

Vitamin D has been demonstrated to have an impact on several human organs and systems in addition to maintaining the equilibrium of calcium and phosphate. Numerous organ systems are influenced by the pleiotropic effects of vitamin D. It controls bone mineralization, muscular function, and calcium and phosphate balance in the musculoskeletal system. It decreases B-cell proliferation and autoantibody synthesis, boosts regulatory T-cell activity, and inhibits pro-inflammatory Th1 and Th17 responses in the immune system [37,38]. Vitamin D affects the renin–angiotensin–aldosterone pathway [39], decreases arterial stiffness, and modifies endothelial function in the cardiovascular system [40]. It influences insulin sensitivity and pancreatic β-cell activity in the endocrine system, and a deficit is associated with a higher risk of type 2 diabetes [19]. By regulating neurotransmission, oxidative stress, and neuroinflammation, vitamin D exerts neuroprotective effects inside the neurological system [41,42,43]. Lastly, although it is still poorly characterized, observational data point to functions in cancer biology [44].

### 2.2. Implications of Vitamin D in Immunomodulation

1,25-dihydroxycholecalciferol (1,25(OH)_2_D_3_, calcitriol), the active form of vitamin D, is essential for controlling both innate and adaptive immunity. Extrarenal conversion takes place in immune cells including monocytes, dendritic cells, and macrophages, particularly in inflammatory regions, in addition to being renally activated by CYP27B1. Direct immunomodulation in lymphoid tissues is made possible by this local synthesis, which has paracrine and autocrine effects that are not dependent on calcium–phosphate metabolism [45,46].

By encouraging Toll-like receptor-mediated responses and the generation of antimicrobial peptides like cathelicidin, vitamin D improves the antimicrobial capability of immune cells in innate immunity. In addition, calcitriol reduces excessive inflammation by suppressing the release of pro-inflammatory cytokines (such as TNFα, IL-1, IL-6, IL-12, and IL-8), decreasing the maturation of dendritic cells and the expression of MHC class II/co-stimulatory molecules, and changing the polarization of macrophages to the anti-inflammatory M2 phenotype. While minimizing tissue damage, this delicate balance between activation and repression aids in the removal of pathogens [35,47,48,49].

Vitamin D influences T- and B-cell responses in adaptive immunity. It promotes the growth and activity of regulatory T cells (Tregs), raises Treg/CD3 ratios, and restores self-tolerance while suppressing dendritic cell-driven antigen presentation, which lowers effector T-cell activation. Additionally, vitamin D promotes a balanced Th1/Th2 profile while suppressing Th1 and Th17 responses, which are important causes of autoimmunity. RORα/γ-dependent mechanisms that limit Th17 cell differentiation mediate additional regulatory effects [50,51,52,53].

The intracellular vitamin D receptor and 1,25(OH)_2_D regulate over 900 genes which are involved in the innate and adaptive immune system as well as several physiological processes. Thus, a lack of vitamin D has been linked to a variety of illnesses, such as metabolic, neuromuscular, and cardiovascular conditions, hypertension, neoplasms, and autoimmune and infectious diseases. Regarding the innate response, it has been demonstrated that vitamin D stimulates natural killer (NK) T cells, which in turn modulates the release of cytokines like IFN-alpha and IL-4. At the same time, macrophage activation is suppressed and M2 “anti-inflammatory” macrophages are dominant [54,55,56].

Vitamin D decreases the development of memory B-cells, inhibits the proliferation and differentiation of B cells into plasma cells, and lowers the release of immunoglobulins (IgA, IgG, and IgM), while its effects on IgE are still unclear. When taken together, these processes show how vitamin D deficiency may lead to increased vulnerability to infections and autoimmunity, decreased tolerance, and exacerbated inflammation [45,57].

### 2.3. Vitamin D Immunomodulation in SLE Patients

Vitamin D has emerged as a pivotal immunomodulatory factor with regulatory actions on both innate and adaptive immune compartments. The vitamin D receptor, which is expressed by a variety of immunocytes, such as dendritic cells, macrophages, and lymphocyte subsets, mediates its biological action.

Dendritic cells (DCs) exposed to 1,25-(OH)_2_D_3_ during early development in the innate immune system have a tolerogenic phenotype, which is typified by increased IL-10 production and decreased IL-12 production. This cytokine profile promotes the production of regulatory T cells (Tregs) while suppressing allogeneic T-cell activation [51].

Because vitamin D’s effects on adaptive immunity vary depending on the T-cell subset, they are similarly complex. Responses from CD4+ T cells, which express VDR when activated, are subset-specific. Vitamin D can partially inhibit Th1 cells by downregulating IL-2 and IFN-γ, even when VDR expression is quite low. The Th2 and Th17 subgroups show stronger impacts. As demonstrated in experimental autoimmune encephalomyelitis models, 1,25-(OH)_2_D_3_ increases IL-4 production in Th2 cells through GATA3 and STAT-6 signaling, strengthening their capacity to counteract Th17-driven inflammation. Vitamin D strongly inhibits Th17 cells, which are increasingly linked to the pathophysiology of SLE, rheumatoid arthritis, and multiple sclerosis. This inhibition occurs on several levels: Th17 plasticity is limited into more pathogenic “non-classical” Th1-like cells that co-express IFN-γ and IL-17; Th17 differentiation is blocked by inhibiting transcription factors like RORC and CCR6. These pathways are supported by clinical data in vitamin D-deficient lupus patients, which show that supplementation increases Treg proportions while decreasing Th1/Th17 frequencies, stabilizing disease activity without increasing immunosuppressive medication [58,59,60,61].

Another important target of vitamin D is regulatory T cells. 1,25-(OH)_2_D_3_ upregulates co-inhibitory molecules such CTLA-4 and PD-1, boosts IL-10 production, and stimulates FoxP3 expression in both animal and in vitro models. The FoxP3 promoter’s VDR binding regions offer a direct transcriptional pathway for these actions. Additionally, vitamin D increases the ability of Tregs to suppress through the activation of indoleamine 2,3-dioxygenase, whereas a lack of it hinders the ability of Tregs to migrate in lupus. All of these results highlight the ability of vitamin D to change the balance of CD4+ T cells from pro-inflammatory Th1/Th17 subsets to anti-inflammatory Th2 and Treg populations [54].

Despite being less researched in lupus, CD8+ T cells also show a strong response to vitamin D. Compared to CD4+ cells, they express more VDR, and when VDR-deficient CD8+ T cells are experimentally transferred, severe colitis is induced along with increased production of IFN-γ and IL-17, especially when IL-10 is not present. 1,25-(OH)_2_D_3_ treatment decreases the quantity of hyperactivated CD8+ T cells and the pro-inflammatory cytokines they secrete [62]. Clinical similarities have been observed in psoriasis, a CD8-dominant autoimmune illness, where topical vitamin D analogs are well-established treatments [63].

When combined, these results demonstrate vitamin D’s versatility as an immune response regulator. Vitamin D creates a cytokine milieu that promotes immunological tolerance over autoimmunity by promoting tolerogenic dendritic cells, modifying macrophage polarization, inhibiting pro-inflammatory Th1/Th17 and CD8+ subsets, and enhancing Th2 and Treg activity. Its therapeutic value in conditions like SLE, where chronic inflammation is caused by an imbalance between effector and regulatory immunity, is further supported by these coordinated actions. Due to the fact that B cells act as antigen-presenting cells and generate antibodies linked to lupus, they are pathologically significant in SLE. Very little research has been done on the precise function of vitamin D in lupus B cells. However, the fact that the VDR binds to the VDRE in lymphoblastoid B cell populations provides evidence of the role that vitamin D plays in B cells with relation to the physiological regulation of inflammatory responses. According to the findings thus far, 1,25-(OH)_2_D_3_ appears to have two separate actions, depending on the B cell’s stage of development. According to studies, 1,25-(OH)2D3 caused apoptosis and reduced B cell proliferation and immunoglobulin (Ig) class switching. By interfering with nuclear NF-B translocation and CD40 co-stimulation, vitamin D prevents B cell development. On the other side of the B cell proliferation distribution, 1,25-(OH)2D3 promotes the migration of plasma cells to inflammatory mucosal surfaces by inducing CCR10 receptors and stimulating the formation of plasma cells from fully differentiated B cells. VDR binds to the IL-10 promotor and increases IL-10 synthesis, possibly reducing autoimmunity, whereas vitamin D has been demonstrated to decrease the formation of lupus-associated autoantibodies, like anti-nuclear antibody (ANA), irrespective of its effect on B cell differentiation [64,65,66].

### 2.4. Epigenetic Dysregulations in Vitamin D-Deficient SLE Patients

The most researched autoimmune disease linked to epigenetic changes is thought to be SLE. Mazzone et al. discussed the significance of epigenetic changes in autoimmune diseases, including SLE patients, in their extensive review [67]. The methylation status of several genes in T cells, including CD11a (ITGAL), perforin (PRF1), CD70 (TNFSF7), and CD40LG (TNFSF5), has been highlighted as a contributing component to the pathophysiology as well as progression to SLE. Compared to DNA methylation, changes to histone patterns as well as the function of noncoding RNAs in SLE have received less attention [68].

Yang et al. have conducted a meta-analysis to assess the effect of VDR gene polymorphisms on the risk of development of SLE. According to the researchers, correlations have been shown between polymorphisms and various populations: the VDR ApaI polymorphism and the general population’s susceptibility, the VDR BsmI polymorphism and the African and Caucasian populations’ susceptibility, and the VDR FokI polymorphism and the African population’s susceptibility [69]. Together with the TaqI polymorphism, the VDR gene has four polymorphic sites, including the previously known ApaI, BsmI, and FokI polymorphisms. The gene’s intron region contains the ApaI and BsmI sites, whereas exon-2’s FokI creates an alternative transcription start site and exon-9’s TaqI results from a silent T to C change. The metabolic pathways for vitamin D can be disrupted by any of the aforementioned locations [23,70].

Even though the cited study found no evidence of a significant association between TaqI polymorphisms and SLE susceptibility in the groups studied, more research is necessary to support this finding. Due to the prevalence of vitamin and mineral deficiencies in this patient population, dietary treatments in SLE are a commonly studied issue. Appropriate supplementation can prevent or lessen the severity of the condition since it has immunomodulatory, anti-inflammatory, and antioxidant actions [69,70].

## 3. Research Methods

A structured literature search was conducted to identify relevant studies addressing the role of vitamin D in systemic lupus erythematosus. Searches were performed in PubMed/MEDLINE, Scopus, and Web of Science databases, covering the period from January 2010 to August 2025. The following keywords and Medical Subject Headings (MeSH) terms were used in various combinations: “systemic lupus erythematosus”, “SLE”, “vitamin D”, “25-hydroxyvitamin D”, “1,25-dihydroxyvitamin D”, “autoimmunity”, “immune modulation”, “autoantibodies”, “disease activity”, “nephritis”, “cardiovascular”, “infections” and “neurolupus”. Boolean operators (AND, OR) were applied to refine the search.

Inclusion criteria comprised original research articles, clinical trials, meta-analyses, systematic reviews, conference abstracts, and relevant observational studies published in peer-reviewed journals. Studies were considered eligible if they reported serum vitamin D levels in SLE patients, examined associations with disease activity, autoantibody profiles, or clinical manifestations, or investigated mechanistic insights into immunomodulatory effects of vitamin D relevant to lupus.

Exclusion criteria included case reports, editorials, and studies not directly addressing vitamin D or SLE. References of retrieved articles were screened manually to identify additional relevant studies. Priority was given to articles published in the last ten years, although older landmark studies were also considered when necessary for mechanistic or historical context.

## 4. SLE Disease Activity and Hypovitaminosis D

Clinicians usually urge SLE patients to use sunscreen and limit their exposure to sunlight because it is one of the most powerful triggers for SLE flares. Low vitamin D in SLE patients is also caused by the effects of medications including calcineurin inhibitors, anticonvulsants, and glucocorticoids, as well as impaired 1-hydroxylation of 25-(OH)D_3_ as a result of renal insufficiency.

The association between vitamin D and SLE has been documented for forty years. The earliest study, which was published in the late 1970s, stated that low blood 1,25-(OH)_2_D_3_ levels were present in seven out of twelve pediatric SLE patients [71]. The association between hypovitaminosis D and the occurrence of SLE was subsequently validated by many larger-scale case–control studies, especially in patients with lupus nephritis [72].

Irfan et al. (2022) [73] conducted a comprehensive review and meta-analysis of six randomized controlled trials with a total of 276 patients to assess the impact of vitamin D supplementation in systemic lupus erythematosus. Autoantibody titers (anti-dsDNA), complement proteins (C3 and C4), patient-reported fatigue, and disease activity as determined by the systemic lupus erythematosus disease activity index (SLEDAI) were the main outcomes evaluated. After supplementation with vitamin D, the pooled analysis showed a substantial decrease in disease activity. A standardized mean difference (SMD) of −0.85 (95% CI −1.12 to −0.58; *p* < 0.00001; I^2^ = 42%) was seen in the decline of SLEDAI scores. Patients with mild baseline disease activity who received longer supplementation (>24 weeks: SMD −0.76, 95% CI −1.01 to −0.50; *p* < 0.00001; I^2^ = 21%) and patients with higher baseline activity who received shorter courses (<24 weeks: SMD −1.23, 95% CI −1.95 to −0.51; *p* = 0.0008; I^2^ = 54%) showed consistent benefits, according to subgroup analysis. On the other hand, anti-dsDNA antibody levels were not substantially impacted by vitamin D administration. In contrast to C4, which did not achieve statistical significance (*p* = 0.07), C3 demonstrated a significant increase upon supplementation (*p* = 0.006). The effect of vitamin D on fatigue was less conclusive. While pooled results suggested improvements in certain domains of fatigue, including fatigue during exercise (*p* = 0.02), fatigue interfering with social life (*p* = 0.03), and overall fatigue severity score (*p* < 0.00001), other dimensions such as fatigue affecting responsibilities (*p* = 0.12) and fatigue perceived as a major problem (*p* = 0.29) failed to demonstrate consistent improvements. Overall, the findings suggest that vitamin D supplementation in SLE is linked to a strong decrease in clinical disease activity and an elevation in C3 complement levels, but has no discernible impact on C4 or anti-dsDNA titers. Firm conclusions cannot be drawn since improvements in fatigue seem to be domain-specific and inconsistent across trials. Though bigger, longer-term trials are needed to elucidate its function in modifying autoantibody synthesis, complement activity, and fatigue outcomes, the overall data points to vitamin D supplementation as a potentially helpful adjunct in the therapy of SLE [73].

Fiblia et al. (2022) [74] examined the impact of daily cholecalciferol supplementation on disease activity and quality of life in female patients with systemic lupus erythematosus (SLE) and hypovitaminosis D in a double-blind randomized controlled trial at Cipto Mangunkusumo Hospital in Jakarta. For 12 weeks, 60 participants between the ages of 18 and 60 were randomly assigned to receive either a placebo or oral cholecalciferol 5000 IU/day. The trial was completed by 27 patients in the intervention group and 25 in the placebo group. With mean values of 15.69 ng/mL (range 8.1–28.2) in the intervention arm and 15.0 ng/mL (range 8.1–25.0) in the placebo arm, vitamin D levels at baseline were similarly low in both groups. Serum 25(OH)D concentrations in the cholecalciferol group increased significantly after 12 weeks of supplementation, reaching a mean of 49.90 ng/mL (26–72.1), indicating an average increase of 33.8 ng/mL. On the other hand, the placebo group showed a mean change of 2.5 ng/mL and just a little rise to 17.35 ng/mL (8.1–48.3). There was a significant difference in increase between the groups (*p* < 0.000). Patients who received vitamin D also showed a substantial improvement in disease activity, as determined by the Mexican version of the SLEDAI (MEX-SLEDAI). Scores decreased by an average of 1.29 points, from a baseline median of 2.67 (range 0–11) to 1.37 (0–6). The MEX-SLEDAI in the placebo group dropped by a mean of 0.12, from 2.6 (0–6) to 2.48 (0–6). The statistically significant difference in improvement between the groups (*p* = 0.015) highlights the positive effect of cholecalciferol on clinical disease activity. In contrast, there were no discernible variations in the quality of life outcomes as reported by the patients. Both groups’ baseline scores on the eight LupusQoL areas were largely positive, with median values in the majority of categories over 50. Even though both arms showed slight increases after the intervention, the difference between the groups was not statistically significant. Overall, this research showed that cholecalciferol at a daily dose of 5000 IU for 12 weeks significantly improved the activity of SLE and efficiently corrected vitamin D insufficiency, but it did not result in a discernible improvement in quality of life over the study period [74].

In order to assess the impact of vitamin D supplementation on disease activity in patients with systemic lupus erythematosus who were vitamin D-deficient, Karimzadeh et al. (2017) [75] conducted a randomized, double-blind, placebo-controlled trial. At Al Zahra Hospital in Isfahan, Iran, 90 patients (81 women and 9 males) with blood 25-hydroxyvitamin D concentrations less than 30 ng/mL were recruited. They were evenly divided into two groups: an intervention group (n = 45) and a placebo group (n = 45). High-dose vitamin D_3_ (50,000 IU weekly for 12 weeks and then 50,000 IU monthly for another six months) was administered to patients in the intervention arm, whereas identical placebo capsules were given to the placebo group. With mean values of 17.36 ± 4.26 ng/mL in the intervention arm and 16.78 ± 4.39 ng/mL in the placebo arm, the baseline vitamin D levels were similar in both groups, but not statistically significant (*p* = 0.53). Following therapy, the supplemented group’s serum vitamin D concentrations increased by an average of around 20 ng/mL, to 37.69 ± 5.92 ng/mL (*p* < 0.001). The placebo group, on the other hand, had mean levels of 16.62 ± 4.61 ng/mL after the intervention (*p* = 0.53), indicating no apparent improvement. Supplementation did not result in a meaningful clinical improvement in disease activity, even when vitamin D insufficiency was significantly corrected. In the vitamin D group, SLEDAI scores decreased from 3.09 ± 2.36 at baseline to 1.62 ± 1.25 after the intervention; this difference was numerical but not statistically significant (*p* = 0.39). In a comparable manner, SLEDAI scores in the placebo group dropped roughly from 3.09 ± 1.20 to 1.98 ± 2.47 (*p* = 0.42), with little distinction between the groups. Within the months of follow-up, the authors found that while high-dose vitamin D treatment successfully increases serum 25(OH)D levels in SLE patients with insufficiency, it does not substantially lower disease activity as measured by SLEDAI [75].

A multicenter, randomized, double-blind, placebo-controlled phase II trial was carried out by Aranow and associates (2015) [76] to determine if vitamin D supplementation reduces the interferon (IFN) signature in patients with systemic lupus erythematosus (SLE). 54 SLE patients with vitamin D insufficiency [25(OH)D ≤ 20 ng/mL], high anti-dsDNA antibodies, stable, inactive illness, and an established IFN signature were randomly assigned to receive either a placebo (n = 19), vitamin D_3_ 2000 IU/day (n = 17), or vitamin D_3_ 4000 IU/day (n = 18) for a duration of 12 weeks. There were 54 patients in the modified intent-to-treat (mITT) group. Serum 25(OH)D concentrations were successfully improved by vitamin D administration. At week 12, 33% of patients in the 2000 IU group and 61% of those in the 4000 IU group had reached vitamin D repletion (≥30 ng/mL), whereas none of the placebo-treated patients had. The placebo group’s baseline mean vitamin D levels were 11.3 ng/mL, the low-dose group’s were 11.8 ng/mL, and the high-dose group’s were 13.8 ng/mL. By week 12, supplementation significantly raised these levels when compared to the placebo group (*p* < 0.001). After controlling for baseline scores, disease activity as assessed by the SELENA-SLEDAI remained constant throughout time and did not significantly differ between treatment groups. Furthermore, only two participants, both in the high-dosage group, went from positive to negative in their anti-dsDNA antibody status (as assessed by local laboratories) between screening and Week 12. Other than the musculoskeletal and cardiorespiratory systems, no BILAG A or B scores were found at week 12. The IFN signature persisted at Week 12 in 79%, 94%, and 100% of the mITT population’s placebo, low-dose, and high-dosage individuals, respectively (*p* = 0.047 when comparing the placebo and pooled vitamin D groups). All repleted patients showed an IFN signature at Weeks 6 and 12, demonstrating the durability of the IFN signature throughout the research, regardless of vitamin D3 administration [76].

In order to assess the effectiveness and safety of vitamin D supplementation in patients with systemic lupus erythematosus, Zheng and colleagues (2019) [77] conducted a systematic review and meta-analysis. There were five qualifying RCTs with a total of 490 participants. Vitamin D supplementation considerably raised the level of blood 25-hydroxyvitamin D in comparison to the placebo therapy (5 trials, 490 participants: standard mean difference (SMD) = 2.072, *p* < 0.001). According to the combined findings of two RCTs, vitamin D supplementation reduced fatigue severity scale scores of SLE patients (2 trials, 79 participants: *p* = 0.001). Anti-double-stranded DNA antibody (anti-dsDNA) positivity and SLE disease activity index scores did not differ substantially (4 trials, 223 participants: 3 trials, 361 individuals; SMD = −0.507, *p* = 0.070). The risk ratio between the vitamin D supplementation group and the placebo treatment group was 0.880, *p* = 0.165. Serious side effects linked to vitamin D administration were not identified in any of the evaluated investigations. This meta-analysis concluded that vitamin D treatment is safe, consistently corrects hypovitaminosis D, and may reduce tiredness in people with SLE. Supplementation, however, did not result in appreciable increases in SLEDAI scores [77].

The first randomized, double-blind, placebo-controlled study focusing on the effects of vitamin D supplementation in juvenile-onset systemic lupus erythematosus was carried out by Lima and colleagues (2016) [78]. With stable background treatment, 40 patients aged ≤25 years with illness onset prior to age 16 were randomized to receive oral cholecalciferol 50,000 IU weekly (n = 20) or a placebo (n = 20) for 24 weeks. Age, BMI, organ involvement, use of glucocorticoids and immunosuppressants, disease activity, and tiredness ratings were among the baseline parameters that were similar between groups. With 95% of the population below sufficiency, mean circulating 25(OH)D levels were significantly low at baseline in both arms (19.1 ± 6.4 ng/mL in the supplemented group vs. 19.5 ± 4.5 ng/mL in the placebo group; *p* = 0.82). With 60% of supplemented patients reaching sufficient concentrations (>30 ng/mL), compared to none in the placebo group, 25(OH)D levels increased considerably in the vitamin D arm after 6 months, rising to 31.3 ± 8.7 ng/mL from 16.5 ± 5.8 ng/mL in the placebo group (*p* < 0.001). Supplementing with vitamin D resulted in a considerable improvement in disease activity. While SLEDAI scores in the placebo group worsened from a median of 3 (0–12) to 5.4 ± 4.5, they decreased from a median of 4 (range 0–8) at baseline to 3.0 ± 3.22 at 6 months. The between-group comparison of change favored supplementation (*p* = 0.011). With a mean decrease of 0 (−2 to 1) in the vitamin D group and an increase of 0 (−6 to 3) in the placebo arm (*p* = 0.006), ECLAM scores also improved in the treated group. Additionally, 15% of patients receiving vitamin D turned from anti-dsDNA positive to negative, but the placebo group showed no changes (*p* = 0.03). Between groups, complement levels and proteinuria did not alter. The Kids Fatigue Severity Scale (K-FSS), which assessed fatigue, also showed a notable improvement. The supplemented group’s global fatigue levels dropped to 3.15 ± 1.44, whereas the placebo group’s scores dropped to 4.30 ± 1.33 (*p* = 0.012). Supplemented patients showed significant improvement in a number of domains, including fatigue during exercise (*p* = 0.027), fatigue occurring easily (*p* = 0.003), fatigue with moderate effort (*p* = 0.017), fatigue perceived as a problem (*p* = 0.026), and fatigue interfering with social life (*p* = 0.010). Fatigue impacting social life showed the greatest domain-specific improvement (mean change −0.95 vs. +0.60; *p* = 0.008). There were no significant side effects, and the supplement was well tolerated. Six individuals (two placebo, four vitamin D) complained of mild epigastric discomfort, and neither blood nor urine calcium levels were abnormal. Therefore, there is compelling evidence from this research that high-dose vitamin D supplementation (50,000 IU/week for 6 months) is safe and beneficial in lowering anti-dsDNA seropositivity, decreasing clinical disease activity, and easing tiredness in juvenile-onset SLE [78].

The randomized trials investigating vitamin D supplementation in SLE are highly heterogeneous, with important differences in dose, duration, patient characteristics, and endpoints. Some studies have applied physiological daily doses in the range of 2000 to 5000 IU, whereas others relied on pharmacological regimens of 50,000 IU given weekly. Duration has generally been limited to short-term interventions of 12 to 24 weeks, with only one study extending to nine months. Most enrolled cohorts had relatively low baseline disease activity, and the outcomes varied considerably, ranging from validated clinical indices of activity to immunological biomarkers such as interferon signatures. Results also appear to differ by age group: in pediatric lupus, one randomized controlled trial demonstrated clearer clinical benefit, while in adult SLE the findings are mixed. The overall body of evidence is constrained by methodological limitations, including small sample sizes, single-center designs, short follow-up, and variability in concomitant background therapy (Table 1).

Taken together, the available randomized trials consistently demonstrate that vitamin D supplementation is safe and effective in correcting biochemical deficiency in SLE patients. Clinical benefits are most evident in pediatric populations, where longer duration and higher weekly dosing yielded reductions in disease activity scores and fatigue with signals of serological improvement. In adults, supplementation improves serum 25(OH)D levels reliably but produces only modest or inconsistent changes in disease activity and has not shown reproducible effects on interferon signatures or autoantibody levels. Across all studies, methodological constraints—including heterogeneous dosing, short duration, and variable background therapy—limit definitive conclusions.

## 5. Lupus Nephritis and Hypovitaminosis D

Lupus nephritis represents one of the most severe organ manifestations of systemic lupus erythematosus, contributing significantly to long-term morbidity and mortality. Its pathogenesis reflects a complex interplay of genetic susceptibility, immune dysregulation, and environmental triggers, with chronic inflammation and autoantibody-mediated injury leading to glomerular damage and progressive renal dysfunction. In recent years, attention has turned to vitamin D as both a modulator of immune activity and a potential factor in renal protection. Hypovitaminosis D is highly prevalent among patients with lupus nephritis, owing to photosensitivity and sun avoidance, glucocorticoid therapy, reduced renal hydroxylation capacity, and dietary insufficiency. Beyond its classical role in calcium and bone homeostasis, vitamin D exerts immunoregulatory effects by downregulating proinflammatory cytokines, inhibiting B-cell autoantibody production, and enhancing regulatory T-cell activity—mechanisms directly relevant to the immunopathology of lupus nephritis. Observational studies consistently demonstrate that low serum 25(OH)D levels correlate with higher disease activity, greater proteinuria, and worse renal outcomes, suggesting that hypovitaminosis D may not only be a marker of disease severity but also a modifiable risk factor. This growing body of evidence positions vitamin D deficiency as an important clinical consideration in the management of lupus nephritis and a potential therapeutic target alongside conventional immunosuppression [79,80,81,82].

A cross-sectional prospective observational research was conducted in Assiut, Egypt, by Khairallah and colleagues (2020) [70] to evaluate the connection between lupus nephritis (LN), systemic lupus erythematosus disease activity, and vitamin D status. The study included 66 age- and sex-matched healthy controls and 100 adult SLE. Patients had significantly lower serum vitamin D levels than controls (3.38 ± 2.55 ng/mL vs. 5.36 ± 2.88 ng/mL, *p* = 0.002). In cases of active disease, the deficiency was particularly noticeable. Vitamin D levels were substantially lower in patients with active SLE (SLEDAI > 8, n = 82) than inactive patients (3.00 ± 2.27 ng/mL vs. 5.10 ± 3.19 ng/mL, *p* = 0.02). Likewise, 25(OH)D concentrations were lower in individuals with renal activity than in those without nephritis (2.58 ± 1.82 ng/mL vs. 5.07 ± 3.10 ng/mL, *p* = 0.003). Significant inverse connections between serum vitamin D and renal SLEDAI scores (r = −0.325, *p* = 0.02) and overall SLEDAI scores (r = −0.361, *p* = 0.01) were found by correlation analysis. Low vitamin D was clinically associated with renal symptoms (*p* < 0.001), malar rash (*p* = 0.02), and mucocutaneous involvement (*p* = 0.05). On the other hand, no noteworthy correlations were discovered with anti-dsDNA antibody titers, tiredness, ESR, CRP, C3, or C4. The greatest predictors of vitamin D insufficiency were shown to be lower C3 levels (β = −0.297, *p* = 0.035) and higher SLEDAI scores (β = −0.455, *p* = 0.002) using multivariable regression. The researchers came to the conclusion that vitamin D insufficiency is quite common among SLE patients in Egypt and is linked to both lupus nephritis and global disease activity [72].

In a sizable cohort of patients with systemic lupus erythematosus (SLE), Bae and colleagues (2015, EULAR abstract AB0613) [83] examined the predictive effect of serum vitamin D levels for lupus nephritis flare. 202 patients who attended the Asan Medical Center in Seoul, Korea, between May and July 2013 were recruited for the study. Serum 25(OH)D levels were taken at baseline, and the patients were monitored for a minimum of a year. This cohort’s mean serum 25(OH)D concentration was 18.44 ng/mL, which was somewhat less than the mean of 19.48 ng/mL for the Korean population as a whole. With a mean baseline SLEDAI of 4.46 ± 3.80, the disease activity was comparatively modest to moderate. Out of the entire cohort, 77 patients (38.1%) had lupus nephritis, and 27 patients (13.8%) had a flare-up during follow-up, which was characterized by hematuria (≥5 RBCs/HPF), urine proteinuria (albumin/creatinine ratio ≥ 1000 mg), and/or the requirement to adjust immunosuppressive treatment. Younger age, low 25(OH)D, anti-Sm antibody positivity, decreased complement C3, and hydroxychloroquine exposure were among the possible risk variables for LN flare that were found by univariate analysis (*p* < 0.20). Multivariate logistic regression was used to analyze these variables. Only low vitamin D (*p* = 0.008) and low C3 levels (*p* = 0.034) were shown to be independent predictors of LN flare in the final model. The potential of vitamin D insufficiency as a biomarker for renal flare prediction was supported by the authors’ conclusion that it is a substantial risk factor for lupus nephritis recurrence [83].

An analytical cross-sectional research assessing the incidence of hypovitaminosis D and its association with clinical and renal activity in patients with systemic lupus erythematosus (SLE) who had biopsy-proven lupus nephritis (LN) was published by Yupe and colleagues [84] (ACR Convergence 2024, Abstract #2658). A total of 24 patients who met the 2019 EULAR/ACR categorization criteria for SLE were included, 12 of whom had renal disease and 12 of whom did not have nephritis. Serum 25(OH)D levels < 30 ng/mL were detected in 58% of the population overall, indicating hypovitaminosis D. Patients with LN had a much greater prevalence (75% vs. 42%) than patients without renal disease. With a Pearson correlation value of −0.594 (*p* = 0.042), vitamin D concentrations within the LN subgroup showed an inverse relationship with 24-h urine protein excretion, indicating that higher proteinuria was linked to more severe vitamin D insufficiency. All LN patients (100%) had interstitial inflammation and glomerular basement membrane rupture, according to histopathological study of renal biopsies; 91.7% also had endocapillary hypercellularity. Nevertheless, there was no clear correlation between vitamin D levels and any of these particular histological characteristics. The degree of proteinuria is a significant predictor of vitamin D insufficiency within LN, the authors observed, and hypovitaminosis D is more common in SLE patients with lupus nephritis than in those without renal involvement. These results support the idea that hypovitaminosis D may be a reflection of renal activity and the loss of vitamin D-binding protein in proteinuric conditions, in addition to being frequent in lupus nephritis [84].

A comprehensive retrospective research evaluating the association between vitamin D level, disease activity, and renal involvement in childhood-onset systemic lupus erythematosus (SLE) was carried out in China by Jiang and colleagues in 2023 [85]. 109 age- and sex-matched healthy controls and 168 patients with initial-onset SLE in childhood (mean age 11.1 ± 2.4 years, 82.7% female) were included in the research. Compared to controls, SLE patients had substantially lower serum 25(OH)D levels (18.63 ± 5.32 ng/mL vs. 25.53 ± 7.02 ng/mL, *p* < 0.05). Deficit (<12 ng/mL) and insufficiency (12–20 ng/mL) were significantly linked to increased disease activity, according to stratification by vitamin D status. SLEDAI-2K scores were lower (median 9.0) for children with sufficient vitamin D levels (≥20 ng/mL) than for those with insufficiency (median 14.5) or deficiency (median 14.0) (*p* < 0.05). Hypovitaminosis D was significantly correlated with renal involvement. Overall, lupus nephritis (LN) affected 91 out of 168 children, or 54.2%. LN prevalence was significantly greater in the vitamin D-deficient and -insufficient groups (71.4% and 68.4%, respectively) than in the sufficient patient group (27.7%, *p* < 0.001). LN patients had considerably lower mean vitamin D levels (17.16 ± 4.85 ng/mL) than non-LN patients (20.37 ± 5.35 ng/mL, *p* < 0.001). Furthermore, vitamin D levels had a positive correlation with GFR (r = 0.237, *p* = 0.002) and complement C3 (r = 0.233, *p* = 0.002) and a negative correlation with 24-h proteinuria (r = −0.384, *p* < 0.001) and SLEDAI (r = −0.244, *p* = 0.001). Histopathological data from 46 biopsied LN patients showed mean 25(OH)D levels by class—type II 18.07 ± 4.50 ng/mL, type III 16.45 ± 4.37 ng/mL, type IV 16.19 ± 3.59 ng/mL, and type V 12.27 ± 3.53 ng/mL; the overall comparison across classes was not statistically significant (*p* = 0.073). Vitamin D levels were lowest in children with type V LN and were significantly lower than in non-type V patients (16.69 ± 3.90 ng/mL; *p* = 0.016). The predictive value of vitamin D in conjunction with other biomarkers was further illustrated by ROC curve analysis. Anti-dsDNA had an area under the curve (AUC) of 0.683, vitamin D had an AUC of 0.705, and C3 had an AUC of 0.754. When the three were taken together, the AUC was 0.803, indicating that low vitamin D improves the LN diagnostic model. According to the authors, vitamin D deficiency and insufficiency are quite common in SLE that develops in children, are associated with higher activity of global and renal diseases, and may be a risk factor for lupus nephritis, particularly type V disease [85].

The majority of the research that is currently accessible is observational, making it vulnerable to reverse causality and confounding. Low 25(OH)D is as much a sign of the severity of the condition as it is a cause, since it can be a result of photosensitivity, decreased sun exposure, glucocorticoid usage, obesity, or the loss of vitamin-D-binding protein in the urine in proteinuric states. Concerns over test technique and the failure to account for seasonal, dietary, and pharmaceutical variations are raised by reported blood levels, which can fluctuate greatly and appear abnormally low in certain cohorts. Strong renal endpoints (eGFR decrease, histologic activity) are seldom evaluated, and study populations are often small, single-center, and have brief follow-up periods. There is also variation in outcome definitions, with some using global indices (SLEDAI) and others using renal surrogates such proteinuria. Although they are still observational, pediatric data show greater associations. Crucially, there is currently no randomized study examining the potential effects of vitamin D supplementation on renal outcomes in lupus nephritis.

Decisive evidence will require prospective, adequately powered RCTs in LN that (i) stratify by baseline 25(OH)D and proteinuria, (ii) standardize dosing/formulation (e.g., cholecalciferol vs. calcifediol), (iii) measure vitamin-D-binding protein and free 25(OH)D alongside total levels, (iv) prespecify renal primary endpoints (proteinuria remission, eGFR slope, histologic activity/chronicity), and (v) rigorously control for seasonality, sun exposure, and background immunosuppression. Until such trials are available, vitamin D in LN should be framed as a corrective adjunct for deficiency, not a proven disease-modifying therapy.

## 6. Cardiovascular Involvement and Hypovitaminosis D in SLE

Cardiovascular involvement is a major contributor to morbidity and premature mortality in systemic lupus erythematosus, encompassing a wide spectrum from accelerated atherosclerosis and endothelial dysfunction to myocarditis, pericarditis, and conduction abnormalities. Traditional risk factors only partially account for this burden, as chronic systemic inflammation, autoantibody-mediated vascular injury, and metabolic alterations significantly amplify cardiovascular risk in SLE. In recent years, hypovitaminosis D has emerged as a potential modifier of this risk profile. Experimental and clinical data suggest that low vitamin D levels promote endothelial activation, vascular stiffness, dyslipidemia, and heightened inflammatory responses, all of which accelerate atherogenesis in SLE. Observational studies indicate that SLE patients with vitamin D deficiency exhibit higher carotid intima-media thickness, increased arterial stiffness, and more frequent cardiovascular events compared with vitamin D-sufficient patients. These findings highlight hypovitaminosis D as both a potential biomarker of heightened cardiovascular risk and a modifiable factor in the comprehensive management of SLE-related cardiovascular disease (Table 1) [86,87].

In a large, multinational inception cohort of patients with systemic lupus erythematosus (SLE), Lertratanakul and colleagues (2014) [88] assessed the association between incident cardiovascular events, cardiovascular risk factors, and serum 25-hydroxyvitamin D levels. 875 individuals from the Systemic Lupus International Collaborating Clinics (SLICC) registry, representing 27 sites in 11 countries, were included in the research. Their mean age was 39.3 ± 13.5 years, the average disease duration was 0.5 ± 0.4 years, and they were primarily female (89.8%). When the patients were included, 72.3% of them had vitamin D insufficiency, with mean 25(OH)D levels of 23.8 ± 13.4 ng/mL. Compared to patients in the highest quartile (Q4: 31–91 ng/mL; mean SLEDAI-2K 4.7; *p* < 0.001), those in the lowest quartile (Q1: 4–13 ng/mL) showed greater disease activity (mean SLEDAI-2K 7.0). More corticosteroid use was also associated with low vitamin D status: at baseline, 79.5% of Q1 patients were taking glucocorticoids, compared to 57.9% in Q4. Logistic analysis showed that increased vitamin D levels were linked to considerably decreased probabilities of hypertension and hyperlipidemia when considering cardiovascular risk factors. For in-stance, regardless of age, sex, BMI, race, nation, or season, patients in Q4 had a lower risk of hypertension (adjusted OR 0.49, 95% CI 0.31–0.77) and hyperlipidemia (adjusted OR 0.50, 95% CI 0.28–0.87) than those in Q1. Diabetes mellitus was not linked to vitamin D. After adjusting for BMI, individuals with low vitamin D continued to have greater levels of inflammatory activity as shown by C-reactive protein. 32 cardiovascular events, including myocardial infarction (n = 7), angina (n = 6), peripheral vascular disease (n = 4), congestive heart failure (n = 4), transient ischemic attack (n = 7), and stroke (n = 4), occurred over a mean follow-up of 5.7 ± 3.0 years. Although there was no significant association between baseline vitamin D quartiles and the incidence of cardiovascular events, the adjusted Cox models showed a non-monotonic pattern: compared with Q1, hazard ratios were 1.15 (95% CI 0.46–2.84) for Q2, 0.68 (0.21–2.13) for Q3, and 0.63 (0.20–1.97) for Q4. Event counts were Q1: 9, Q2: 13, Q3: 5, and Q4: 5. The researchers came to the conclusion that hypovitaminosis D is linked to both cardiovascular and immunological risk profiles since low baseline 25(OH)D levels in SLE patients are independently linked to hyper-tension, hyperlipidemia, increased CRP, and higher SLEDAI-2K scores. Higher vitamin D levels were associated with a trend toward a lower incidence of cardiovascular events, albeit this trend was not statistically significant. This suggests that having appropriate vitamin D status in SLE may have long-term cardiovascular advantages [88].

The association between vitamin D level, disease activity, vascular risk factors, and subclinical atherosclerosis in Chinese patients with systemic lupus erythematosus (SLE) was examined in a major cross-sectional study carried out in Hong Kong by Mok and colleagues (2012) [89]. 290 consecutive SLE patients, 94% of whom were female, were included in the research. Their mean age was 38.9 ± 13.1 years, and their mean illness duration was 7.7 ± 6.7 years. Nearly all patients (96%) had vitamin D deficiency (<30 ng/mL), and 27% had severe deficiencies (<15 ng/mL). Serum 25(OH)D3 levels were 19.1 ± 6.2 ng/mL on average. Autoimmunity and dyslipidemia have been linked to vitamin D insufficiency. Patients with deficiencies had significantly higher atherogenic lipid ratios (total/HDL cholesterol 3.96 vs. 3.07, *p* = 0.02; LDL/HDL cholesterol 2.36 vs. 1.68, *p* = 0.01), lower HDL cholesterol (1.41 vs. 1.59 mmol/L, *p* = 0.004), higher triglycerides (1.69 vs. 1.27 mmol/L, *p* = 0.007), and higher LDL cholesterol (2.96 vs. 2.52 mmol/L, *p* = 0.02). Additionally, their likelihood of having an antiphospholipid antibody was higher (57% vs. 39%, *p* = 0.007). 58 (44%) of the 132 individuals with vascular risk factors who had imaging had subclinical atherosclerosis, which was indicated by abnormal coronary calcium scores or carotid intima-media thickness. Vitamin D levels in individuals with and without subclinical atherosclerosis were not statistically significantly different. In contrast, age (50.1 vs. 37.3 years, *p* < 0.001), postmenopausal status (56% vs. 28%, *p* = 0.002), BMI (23.5 vs. 21.8, *p* = 0.02), and triglyceride levels (1.57 vs. 1.17 mmol/L, *p* = 0.005) were more significantly linked to atherosclerosis. In summary, adverse lipid profiles and antiphospholipid antibody positivity are associated with vitamin D insufficiency, indicating a potential involvement in regulating cardiovascular risk. Subclinical atherosclerosis, on the other hand, was not directly linked to vitamin D deficiency; instead, it was explained by conventional risk factors [89].

The association between serum and dietary vitamin D levels and cardiometabolic risk factors in SLE was investigated by Ruiz-Ballesteros and associates in 2024. Serum calcidiol [25(OH)D] concentrations, vitamin D dietary consumption (as determined by three 24-h food records), and adherence to dietary patterns (DPs) determined by principal component analysis were assessed in 224 Mexican SLE patients and 201 healthy controls (HS) in this cross-sectional investigation. Composite cardiometabolic indices and biochemical measurements were used to evaluate cardiometabolic state. Deficiency in calcidiol (<20 ng/mL) was prevalent and linked to negative metabolic repercussions. Deficiency increased the probability of excess weight by BMI > 25 kg/m^2^ by 1.66 times (*p* = 0.02), low HDL-C (<40 mg/dL) by 2.25 times (*p* < 0.001), and hypertriglyceridemia (≥150 mg/dL) by 1.74 times (*p* = 0.02) in SLE patients. Similarly, a 1.92-fold increased risk of an non-healthy waist circumference (>80 cm) (*p* < 0.01), a 2.05-fold increased risk of an android waist-to-hip ratio (≥0.85) (*p* < 0.01), and a 1.72-fold increased risk of being overweight (*p* = 0.02) were all associated with inadequate vitamin D dietary intake. Non-adherence to a diet plan high in vitamin D food sources (including fish, dairy, and fortified goods) was linked to increased waist circumference, a larger waist-to-hip ratio, higher triglycerides, and lower HDL-C, according to an analysis of dietary patterns. It’s interesting to note that in healthy individuals, the risk of calcidiol deficit increased 2.11 times when this vitamin D-rich dietary pattern was not followed. The study concluded that an adverse cardiometabolic profile in SLE, including dyslipidemia, central obesity, and excess weight, is substantially correlated with both blood vitamin D insufficiency and low dietary vitamin D consumption [90].

Confounding and reverse causality are limitations of the observational design of the cardiovascular investigations. Rather than being a direct cause, lower 25(OH)D levels might be a result of systemic inflammation, obesity, photosensitivity, decreased outdoor exercise, or corticosteroid exposure. Although vitamin D insufficiency was associated with increased disease activity and a higher frequency of hypertension and hyperlipidemia in the SLICC cohort, there were few cardiovascular events and no statistically significant correlations between the two variables. Deficiency was almost universal in the Hong Kong study, which decreased discriminatory power; vitamin D deficiency was associated with antiphospholipid antibody positivity and dyslipidemia, but traditional risk factors like age, menopausal status, and BMI were a better way to explain subclinical atherosclerosis. Although the Mexican cohort’s cross-sectional design precludes establishing directionality, it did include dietary data that demonstrated concordant relationships between poor consumption, low blood vitamin D, and unfavorable metabolic profiles. Measurement variability, small event counts, endpoint heterogeneity, and some studies’ failure to account for seasonality or diet all compromise the capacity to draw conclusions about causality across all cohorts (Table 2).

## 7. Neurolupus and Hypovitaminosis D

A significant contributor to lupus-related morbidity and death, neuropsychiatric SLE (NPSLE) is typified by a wide range of symptoms, such as headache, mood disorders, seizures, psychosis, and cognitive impairment. The pathogenesis of NPSLE is mechanistically linked to vascular disease, complement activation, proinflammatory cytokines, autoantibody deposition, and disruption of the blood–brain barrier [100].

Vitamin D insufficiency is often reported in SLE and has been connected to disease activity, neuropsychiatric symptoms, and cognitive impairment. With receptors extensively expressed in brain areas such the hippocampus, cortex, basal ganglia, and cerebellum, the review highlights that vitamin D serves as a neurosteroid and immunomodulator in addition to maintaining calcium homeostasis. Inhibiting B-cell proliferation and antibody production, increasing regulatory T-cell activity, lowering the expression of proinflammatory cytokines (IL-1, IL-6, IL-17, and TNF-α), and promoting anti-inflammatory cytokines like IL-10 are all effects of active vitamin D (1,25(OH)_2_D_3_). Vitamin D has neuroprotective and remyelination qualities in the central nervous system (CNS), lowers oxidative stress, increases the production of neurotrophic factors, and controls calcium signaling [100,101].

Sultana et al. (2022) [102] conducted a single-center retrospective series of 19 patients with neuropsychiatric systemic lupus erythematosus and found overall low serum 25-hydroxyvitamin D concentrations (mean 18.7 ± 9.8 ng/mL), with 63.2% classified as deficient, 15.8% insufficient, and 21% normal; cognitive performance by the Mini-Mental State Examination (MMSE) averaged 24.2 ± 1.6, and 68.4% met criteria for mild cognitive impairment. On brain perfusion single-photon emission computed tomography (SPECT) analyzed with the easy Z-score imaging system (eZIS), hypoperfusion was present in 78.9%—predominantly in the frontal lobes (superior, middle, inferior), followed by parietal cortex, precuneus, cingulate gyri, and less often basal ganglia, temporal lobe, or cerebellum—and eZIS z-scores grouped as 31.6% normal (mean z 0.52 ± 0.2), 52.6% mild deficit (1.72 ± 0.2), and 15.8% moderate (2.33 ± 0.2). Vitamin D level correlated strongly and inversely with perfusion deficit (25-hydroxyvitamin D vs. eZIS z-score r = −0.896, *p* < 0.001) and positively with cognition (25-hydroxyvita amin D vs. MMSE r = 0.893, *p* < 0.001), while perfusion deficit correlated inversely with cognition (MMSE vs. eZIS z-score r = −0.943, *p* < 0.001); median MMSE decreased across eZIS categories (26 in normal, 24 in mild, 22 in moderate; *p* ≤ 0.005), and median 25-hydroxyvitamin D similarly declined (32 ng/mL in normal, 16.5 in mild, 8 in moderate; *p* ≤ 0.006), with lower vitamin D in those with mild cognitive impairment than those without (12 vs. 32 ng/mL; *p* = 0.001). Voxel-based regression (Statistical Parametric Mapping) identified significant associations between serum 25-hydroxyvitamin D and regional perfusion in bilateral upper frontal regions (middle/superior frontal gyri), left temporal pole, cingulate/precuneus, left posterior insula, and left supramarginal gyrus; the authors conclude that lower vitamin D associates with more severe cortical hypoperfusion and poorer cognition in neuropsychiatric lupus and suggest eZIS-derived SPECT metrics may aid in monitoring responses to vitamin D supplementation, while acknowledging the limitations of a small, retrospective cohort [102].

Hussein et al. (2018) [103] investigated the association between serum 25(OH)D_3_ and cognitive function by examining 30 Egyptian SLE patients and 20 matched controls. SLE patients scored poorly on cognitive tests and had considerably lower vitamin D levels (35.6 ± 27.5 vs. 73.9 ± 15.2 ng/mL, *p* < 0.0001). There were decreases in phonemic fluency (17.9 vs. 30.8, *p* < 0.0001), executive function on the Trail Making Test (178 vs. 80 s, *p* < 0.0001), and total verbal memory recall (CVLT-II) (39.6 vs. 53.0, *p* < 0.0001). There was no difference in depression levels across groups, eliminating mood as a significant confounding factor. Additionally, there was no significant correlation between vitamin D levels with memory or fluency scores, but there was a negative correlation between vitamin D concentrations and executive dysfunction (r = −0.399, *p* = 0.03). The researchers came to the conclusion that vitamin D insufficiency may specifically contribute to executive dysfunction in SLE, supporting the potential value of supplements as a supplementary measure to lessen cognitive impairment [103].

Tay et al. (2015) [101] investigated the connection between cognitive deterioration in SLE and blood vitamin D metabolites. Sixty-one SLE patients and sixty-one age- and sex-matched healthy controls participated in this cross-sectional investigation. The Automated Neuropsychological Assessment Metrics (ANAM) were used to measure cognitive function, and the primary outcome was the total throughput score (TTS). The Hospital Anxiety and Depression Scale (HADS) was used to evaluate anxiety and depression. Quantification of serum 25(OH)D_3_ and total 25(OH)D levels was done using tandem mass spectrometry and liquid chromatography. Compared to controls, SLE patients showed substantially worse cognitive ability, with mean TTS values of 322.2 ± 100.9 versus 395.9 ± 83.3 (*p* = 0.004). Compared to 1.6% of controls, 34.4% of SLE patients had cognitive impairment, which was defined as a TTS below 1.5 SD of the control mean (*p* < 0.001). Although there was no significant difference in the groups’ total vitamin D levels, SLE patients were more likely to have a 25(OH)D_3_ deficit (19.7% vs. 3.3%, *p* = 0.003). Importantly, 25(OH)D_3_ deficiency was an independent predictor of decreased TTS (β = −63.7, SE = 27.5, *p* = 0.025) according to a multivariable model that controlled for age, education, gender, ethnicity, mood, SLE activity (SELENA-SLEDAI), disease damage (SLICC/ACR-DI), and cumulative steroid dosage. On the other hand, overall 25(OH)D levels did not predict cognitive function. In controls, age was a better indicator of cognitive deterioration than vitamin D levels. The researchers concluded that vitamin D_3_ is a more reliable biomarker of neurocognitive risk in SLE patients since it is independently linked to decreased cognition in this cohort, as opposed to total vitamin D [101].

Karnopp et al.’s review article from 2024 summarizes data on vitamin D’s involvement in neuropsychiatric systemic lupus erythematosus from both human and animal investigations. The review’s human studies demonstrate that in NPSLE patients, low blood 25(OH)D_3_ levels are associated with both cerebral hypoperfusion and cognitive impairment. Sultana et al. (2022) [102] showed that hypovitaminosis D was linked to decreased brain perfusion on SPECT imaging in NPSLE, Tay et al. (2015) [101] found that 25(OH)D_3_ deficiency is an independent predictor of cognitive impairment, and Hussein et al. (2018) [103] reported lower vitamin D in SLE patients with worse executive function. Studies on animals support these conclusions: Vitamin D-supplemented MRL/lpr and pristane-induced lupus models demonstrated enhanced cognition and increased hippocampal VDR expression; in the PIL model, the review reports a positive VDR–IgG correlation (rather than decreased IgG), alongside evidence consistent with neuroinflammation modulation [104].

The research indicates that while supplementing has neuroprotective and immunomodulatory benefits in preclinical models, hypovitaminosis D is closely linked to neuropsychiatric symptoms and cognitive impairments in lupus. The danger of toxicity at high doses necessitates a thorough evaluation of the best dosage regimens, as clinical data is still limited and inconsistent. To elucidate causation, establish supplementation guidelines, and get a deeper understanding of the dual influence of vitamin D on central and peripheral nervous system involvement in lupus, more longitudinal and interventional research is recommended. The studies on human and animal models, as well as reviews and ongoing clinical trials and outcomes, are presented in Table 3.

## 8. Risk of Infections in SLE and Hypovitaminosis D

High morbidity and death rates are linked to infections, which are prevalent in SLE patients. This predisposition may be partially caused by immunosuppressive therapy and immune system abnormalities linked to SLE, which make patients more prone to infection. The correlation between hypovitaminosis D and SLE has not been that researched in the literature [109].

The impact of baseline blood vitamin D on COVID-19 severity, recovery, and tiredness in individuals with SLE was investigated in Adel et al.’s 2022 study [110]. Included were 38 SLE patients with suspected or confirmed COVID-19 (mean age 49.2 ± 8.1 years). 42.1% had vitamin D deficiency (≤20 ng/mL), 23.7% had insufficiency (21–29 ng/mL), and 34.2% had normal levels (≥30 ng/mL) upon diagnosis. In comparison to those with normal vitamin D, patients with low vitamin D (insufficiency + deficiency) had lower lymphocyte counts (1080 vs. 1931/cm^3^, *p* < 0.05), higher ESR (52.6 vs. 35.9 mm/h, *p* < 0.05), a longer disease duration (6.1 vs. 3.8 years, *p* = 0.061; trend), and a longer recovery time from COVID-19 (22.6 vs. 18.5 days, *p* < 0.05). Clinically, compared to 30.8% in the group with normal vitamin D, 68% of patients with low vitamin D had significant COVID-19 symptoms (*p* < 0.05). For outcomes, 5 of 7 patients who were non-survivors or lost to follow-up (71.4%) were from the low-vitamin-D group, and baseline vitamin D was nominally lower in the non-survivor/lost to follow up group vs. survivors (17.2 vs. 23.6 ng/mL; *p* ≈ 0.06), so interpret mortality differences cautiously. Repeat testing after recovery revealed that most individuals who had been low in vitamin D had returned to normal levels as a result of taking supplements. However, individuals with low baseline vitamin D levels (60%) experienced post-COVID fatigue more frequently than those with normal levels (36.3%). Overall, adequate vitamin D was associated with milder acute illness, faster recovery, and less post-COVID fatigue; associations with mortality were suggestive rather than definitive [110].

In their 2021 research, Susanto et al. investigated the connection between SLE patients’ innate immune activity and vitamin D levels. Thirty SLE patients were enrolled, with a mean age of 27.7 ± 10.7 years and 96.7% females. A mean of 9.98 ± 4.64 ng/mL for serum 25(OH)D was found in all, indicating vitamin D deficiency (<20 ng/mL). The expression of Toll-like receptor 2 (TLR2) by CD11b+ immune cells was assessed using flow cytometry on saliva samples. TLR2 expression averaged 26.0 ± 20.9%. Serum 25(OH)D concentration and TLR2 expression on CD11b+ salivary immune cells were shown to be significantly positively correlated by statistical analysis (Spearman r = 0.434, *p* < 0.05). It was confirmed by linear regression that the sole independent predictor of TLR2 expression was vitamin D (*p* < 0.05). In identifying oral infections and triggering antimicrobial peptides like cathelicidin, the authors emphasize the critical function that TLR2 plays. In individuals with SLE, low vitamin D levels may disrupt this system, resulting in decreased pathogen detection and increased vulnerability to oral infections (such as *Candida albicans*). Their conclusion that vitamin D insufficiency is closely linked to decreased salivary expression of innate immune receptors supports the hypothesis that hypovitaminosis D increases the risk of infection in SLE by impairing mucosal immune function [111].

Both studies support the hypothesis that hypovitaminosis D may aggravate infection risk and outcomes in SLE, either through impaired recovery from viral illness or reduced innate immune defense. However, their conclusions are limited by small sample sizes, single-center designs, and reliance on surrogate markers rather than robust clinical endpoints. Overall, the evidence is suggestive but not definitive, highlighting the need for larger, well-powered mechanistic and interventional studies to clarify the role of vitamin D supplementation in infection prevention and recovery among SLE patients.

## 9. Conclusions

Hypovitaminosis D is highly prevalent in SLE and associates reproducibly with higher disease activity, renal involvement, adverse cardiometabolic profiles, and neurocognitive impairment, with convergent mechanistic support from immunology, epigenetics, and animal models. Supplementation reliably corrects deficiency and appears safe; signals of benefit include modest reductions in clinical activity and fatigue and organ-specific improvements in juvenile cohorts, while effects on interferon signatures, complement, and autoantibodies remain inconsistent. The field now needs adequately powered, multicenter randomized trials that standardize vitamin D assays, distinguish 25(OH)D_3_ from total 25(OH)D, and prespecify organ-targeted outcomes (e.g., proteinuria and eGFR for nephritis, carotid measures and events for cardiovascular disease, cognitive batteries with perfusion imaging for NPSLE). Defining therapeutic targets, dosing strategies, and monitoring frameworks—alongside safety surveillance for hypercalcemia—will determine whether vitamin D can move from a biologically plausible adjunct to an evidence-based component of comprehensive SLE care.

## Figures and Tables

**Table 1:** Studies related to disease activity and hypovitaminosis D in SLE patients.

Study (Year)	Population (n, Age)	Mean Baseline 25(OH)D	Intervention vs. Control	Duration	Primary Outcomes	Key Findings	Risk of Bias/Limitations	Overall Appraisal
Aranow 2015 [76]	Adults with SLE, vit D ≤ 20 ng/mL, stable inactive disease (n = 57 randomized—54 intended to treat)	~11–14 ng/mL	Vitamin D_3_ 2000 IU/day or 4000 IU/day vs. placebo	12 weeks	IFN gene signature	Repletion in 33% (2000 IU) and 61% (4000 IU). No reduction in IFN signature; disease activity unchanged.	Small sample; short follow-up; biomarker-centric endpoint; underpowered for clinical change.	Safe correction of deficiency; no effect on IFN signature or activity (SELENA-SLEDAI; anti-dsDNA) at 12 weeks.
Karimzadeh 2017 [75]	Adults with SLE, 25(OH)D < 30 ng/mL (n = 90)	~17 ng/mL	Vitamin D_3_ 50,000 IU weekly ×12 weeks then monthly ×3 months vs. placebo	9 months	SLEDAI	25(OH)D ↑ ~20 ng/mL; SLEDAI decreased numerically but not significant vs. placebo.	Mixed background therapy; modest baseline activity; possible underpowering.	Corrects deficiency; clinical improvement signal weak.
Fiblia 2022 [74]	Adult women with SLE + hypovitaminosis D (n = 52 completed)	~15–16 ng/mL	Cholecalciferole 5000 IU/day vs. placebo	12 weeks	MEX-SLEDAI; LupusQoL	Significant MEX-SLEDAI improvement; no QoL change.	Single-center; female-only; low baseline activity; short duration.	Positive effect on activity over 12 weeks.
Lima 2016 (pediatric) [78]	Juvenile-onset SLE (≤25 yrs; onset <16 yrs) (n = 45)	~19 ng/mL	D_3_ 50,000 IU/week vs. placebo	24 weeks	SLEDAI; ECLAM; anti-dsDNA; fatigue (K-FSS)	SLEDAI & ECLAM improved; fatigue improved; 15% converted anti-dsDNA(+)→(−); safety acceptable.	Small RCT; pediatric only; organ-specific outcomes limited.	Strongest clinical benefit; supports longer duration and higher dosing in pediatric SLE.
Irfan 2022 (meta-analysis) [73]	6 RCTs, n = 276 (mixed adult/pediatric SLE)	Varied (often deficient)	Pooled daily and weekly regimens	12–36 weeks	SLEDAI, C3, C4, anti-dsDNA, fatigue	SLEDAI decreased (SMD −0.85); C3 ↑; no effect on anti-dsDNA or C4; fatigue inconsistent.	Moderate heterogeneity; variable regimens; small included trials.	Suggests modest activity improvement and complement rise; results heterogeneous.
Zheng 2019 (meta-analysis) [77]	5 RCTs, n = 490 (mainly adults)	Varied (often deficient)	Pooled daily and weekly regimens	12–52 weeks	25(OH)D, SLEDAI, fatigue	25(OH)D consistently ↑; fatigue improved; no significant pooled SLEDAI effect; supplementation safe.	Clinical heterogeneity; endpoints diverse.	Supports correction of deficiency and fatigue benefit; uncertain effect on disease activity.

Symbols: ↑—increased; →—converted to.

**Table 2:** Studies related to cardiovascular risk and hypovitaminosis D in SLE patients.

Study	Type	Country	Population	CV Endpoint(s)	Main Findings/Results
Wu et al. (2009) [91]	Cross-sectional	USA (female cohort)	79 women with SLE	CV risk factors	Low vitamin D linked with higher BP, low LDL-cholesterol, but associations were not statistically significant after BMI adjustment
Reynolds et al. (2011) [92]	Cross-sectional	UK	67 SLE	Aortic pulse-wave velocity (aPWV), carotid plaque and intima media thickness	Vitamin D deficiency strongly associated with increased aortic stiffness, independent of classic risk factors
Mok et al. (2012) [89]	Cross-sectional	Hong Kong	290 SLE	Carotid intima media thickness/plaque	Vitamin D associated with dyslipidemia, but not with subclinical atherosclerosis
Kiani et al. (2013) (Hopkins Lupus Cohort) [93]	Prospective cohort	USA	~100 SLE	CAC progression, carotid IMT, hsCRP	Baseline vitamin D did not predict CAC progression, IMT, or hsCRP over 2 years
Lertratanakul et al. (2014) (SLICC inception) [88]	Prospective cohort	Multicenter international	~1000 newly diagnosed SLE	CV risk factors; composite events	Lower vitamin D associated with worse CV risk factors. Trend: higher vitamin D linked to fewer CV events
Jung et al. (2014) [94]	Cross-sectional	Korea	102 SLE and 52 controls	Carotid IMT/plaque	No significant correlation between vitamin D and IMT/plaque or disease activity markers
Robinson et al. (2014) (APPLE substudy) [95]	Pediatric cohort (effect modifier)	USA	201 pediatric SLE	Carotid IMT progression	Subjects with serum 25(OH)D ≥ 20 ng/mL had less mean-max carotid IMT progression following 3 years of atorvastatin treatment
Kamen & Oates (2015) [96]	Interventional (pilot)	USA	9 SLE	Endothelial function—flow-mediated dilation (FMD)	FMD improved in ~50% of vitamin D-repleted patients vs. 0% in non-repleted
Reynolds et al. (2016) [97]	Interventional	UK	40 SLE—2 groups—22 deficient and 18 repleted	Endothelial function (FMD), angiogenic cells	Vitamin D improved FMD and increased circulating angiogenic cells; FMD change correlated with Δ25(OH)D
Mellor-Pita et al. (2019) [98]	Cross-sectional	Spain	47 SLE	Arterial stiffness—carotid femoral pulse wave velocity (PWV)	Patients with arterial stiffness had higher vitamin D levels, most of the patients were on Vitamin D–calcium supplements
Ruiz-Ballesteros et al. (2024) [90]	Cross-sectional	Mexico	224 SLE and 201 controls	Cardiometabolic risk	Low vitamin D associated with higher cardiometabolic risk (HTN, dyslipidemia, obesity)
Yan et al. (2024) [99]	Mechanistic (omics)	China	31 SLE	Lipid metabolism	Vitamin D deficiency associated with atherogenic lipid pathway alterations

**Table 3:** Studies on vitamin D effects in neuropsychiatric systemic lupus erythematosus.

Study	Country	Sample Size	Supplement Duration	Vitamin D Supplement Doses	Outcomes
Tay et al. (2015) [101]	Singapore	61 SLE, 61 controls	Cross-sectional (no supplementation)	N/A (observational)	25(OH)D_3_ deficiency independently predicted worse total throughput score (TTS); SLE patients scored less in TTS than healthy controls
Hussein et al. (2018) [103]	Egypt	30 SLE, 20 controls	Cross-sectional (no supplementation)	N/A (observational)	Lower vitamin D levels in SLE vs. controls; deficiency associated with worse executive dysfunction (TMT-B)
Sultana et al. (2022) [102]	Bangladesh	19 NPSLE	Cross-sectional (no supplementation)	N/A (observational)	Positive correlation between vitamin D levels and hypoperfusion in brain regions related to cognitive functions; vitamin D levels lower in NPSLE with lower clinical mini-mental state examination (MMSE) scores
Yan et al. (2019) [105]	China	20 MRL/lpr mice and 10 C57BL/6 J mice controls	4 weeks	Aerococcus and one daily intraperitoneal injection of Vitamin D (2 μg/kg/day)	Vitamin D therapy improved neurobehavioral abnormalities. At the molecular level, vitamin D treatment regulated the expression of caspase-3 and Bcl-2, triggered the production of VDR, and decreased the quantity of dead cells in the CA1 area of the hippocampus.
Li et al. (2023) [106]	China	40 MRL/lpr mice	3 weeks	1,25(OH)2D3 4 μg/kg intraperitoneal, 2×/week	Delayed choroid plexus infiltration, preserved BCSFB, through activation of PPARγ/NF-κB/TNF-α and inhibition of TGF-β/Smad signaling
Karnopp et al. (2022) [107]	Brazil	23 mice divided into three groups: control (CO), pristane-induced lupus (PIL) and PIL mice supplemented with VD (VD)	6 months	Calcitriol 2 μg/kg every 2 days	Hippocampal IgG infiltration was greater in the PIL group than in the CO group. There was evidence that vitamin D might decrease IgG infiltration. Each group’s hippocampal region was comparable. There were no variations in VDR expression between the groups. VDR and IgG expression in the hippocampus were found to be positively correlated.
Karnopp et al. (2024) [104]	Brazil	Review	N/A	N/A	Hypovitaminosis D linked to NPSLE cognition deficits; supplementation beneficial in animal models
UMIN000056299 (ongoing) [108]	Bangladesh	72 NPSLE patients (planned)	6 months	Vitamin D3 oral supplementation (dose not yet reported)	Primary: cognition (MMSE); Secondary: brain perfusion (SPECT), serum Vit D levels

## Data Availability

Not applicable.
